# Case Report: Unusual radiological findings in adult human metapneumovirus infection

**DOI:** 10.3389/fmed.2025.1687321

**Published:** 2025-10-27

**Authors:** Ling Chieng Jiun, Ho Shuang Yee, Mohd Imree Azmi

**Affiliations:** Department of Radiology, Faculty of Medicine, Universiti Kebangsaan Malaysia, Cheras, Malaysia

**Keywords:** CT, cystic lung disease, adult, human metapneumovirus (hMPV), imaging

## Abstract

Human metapneumovirus (hMPV) is a globally prevalent respiratory pathogen, primarily affecting young children, the elderly, and immunocompromised individuals. Typical airway-centric radiological findings like other viral infections are commonly reported. However, atypical findings in computed tomography (CT) have yet to be reported in literature. We present a case of hMPV infection in an immunocompetent adult with rare findings of cystic lesions in CT thorax. A 67-year-old female with underlying valvular heart disease and chronic heart failure presented to our centre with respiratory symptoms and reduced effort tolerance. Initial chest radiographs were suggestive of an active pulmonary infection superimposed on acute pulmonary edema, leading to empirical antibiotic treatment with the differentials of community-acquired pneumonia. Despite antibiotics and diuretics treatment, her condition worsened requiring oxygenation supplementation. High-resolution computed tomography (HRCT) thorax was performed and demonstrated typical airway-centered findings including peribronchial wall thickening and ground-glass opacities. In addition, multiple cystic lung lesions of varying sizes in random distribution are seen scattered in both lung fields. Reverse transcription polymerase chain reaction (RT-PCR) confirmed an hMPV infection, with no evidence of bacterial or other viral co-infection. The patient improved with supportive care and was discharged after 5 days of admission. While there is no preceding literature on atypical lung changes during the course of hMPV infection, this case highlights the possibility of the broader imaging dynamics of this virus.

## Introduction

Human metapneumovirus (hMPV) is a globally prevalent respiratory pathogen, primarily affecting young children, the elderly, and immunocompromised individuals. Radiologically, hMPV infections typically present an airway-centric pattern that closely resembles other viral infections but also exhibits features overlapping with bacterial pneumonia. These include bronchial wall thickening, centrilobular nodules, peri-bronchial consolidation, ground-glass opacities (GGO), and nodular consolidation (1–4). However, the cystic changes in CT thorax have yet to be reported in literature. Here, we presented a case of a 67-year-old female with a background of valvular heart disease and chronic heart failure, who tested positive for hMPV infection. Radiological findings highlighted atypical findings of multiple cystic lung lesions with typical airway-centered findings of viral infection.

## Case description

A 67-year-old female with a background of valvular heart disease and chronic heart failure presented to our facility with complaints of respiratory symptoms and reduced exercise tolerance over the preceding 2 days. Further history revealed close contact with a symptomatic spouse and non-compliance with her prescribed diuretics medications for the past week.

**Figure 1:**
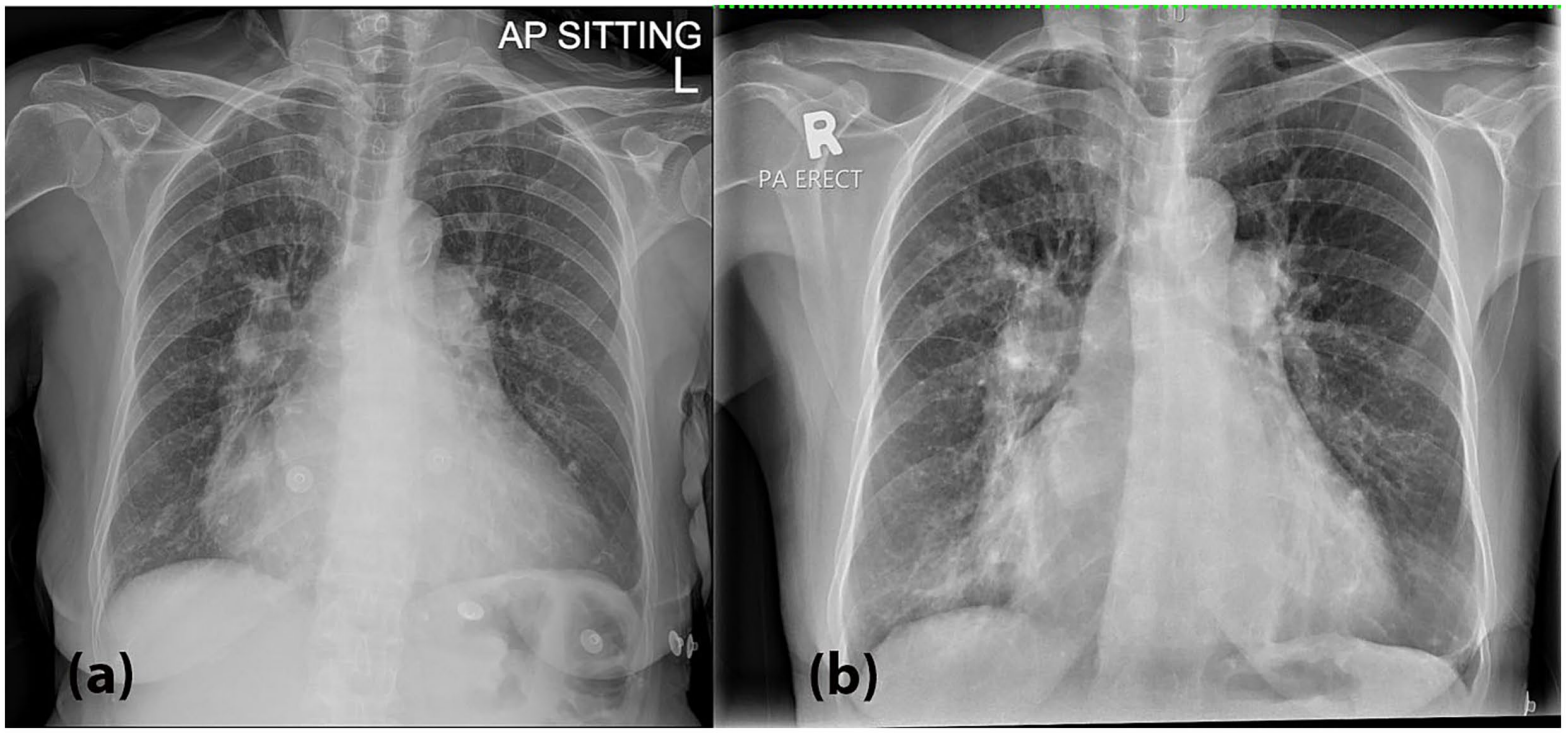
**(a)** A chest radiograph (CXR) performed on presentation demonstrated bilateral hilar opacities with peribronchial cuffing, along with radiographic features suggestive of pulmonary edema, including fluid in the right horizontal fissure and Kerley B lines, especially in the right lower zone. These findings are suggestive of an active pulmonary infection superimposed on acute pulmonary edema. **(b)** A repeat CXR on Day 3 showed worsening bilateral hilar and lower zone opacities, along with persistent peribronchial cuffing.

Upon arrival at the Emergency Department, she appeared breathless but was not using accessory muscles and remained alert. She was febrile (39.6 °C), tachypneic (respiratory rate of 24–26 breaths/min) and hypoxemic with peripheral oxygen saturation of 92% on room air. Her blood pressure and heart rate were unremarkable; BP 119/68 HR 90 bpm. On physical examination, lung auscultation revealed crepitations localized to the right lower lung zone. Arterial blood gas (ABG) analysis demonstrated type 1 respiratory failure, while inflammatory markers including PCT (0.04 ng/mL), CRP (2.52 mg/dL) white blood cell count (6.6 × 10^9^/L) were within normal limits. She tested negative for COVID-19. A chest radiograph (Figure 1a) performed on presentation demonstrated features suggestive of pulmonary edema. In correlation with the clinical presentation and laboratory results, the initial working diagnosis was community-acquired pneumonia (CAP), and the patient was subsequently admitted for empirical antibiotic therapy (intravenous augmentin 1.2 g QID and oral azithromycin 500 mg OD) targeting both typical and atypical respiratory pathogens. Her previous diuretics medications were also on board, which were oral spironolactone 12.5 mg OD and furosemide 20 mg PRN.

During hospitalization, the patient experienced recurrent fever spikes and oxygen desaturation, necessitating anti-pyretic and supplemental oxygen via nasal prong 3 L/min. A repeat chest radiograph on Day 3 (Figure 1b) showed worsening bilateral hilar and lower zone opacities, along with persistent peribronchial cuffing. Laboratory investigations at that point revealed thrombocytopenia and leucocytopenia, with infective screenings (blood and sputum bacterial and tuberculosis cultures, PCT, CRP, dengue serology, BFMP) yielded negative results.

**Figure 2:**
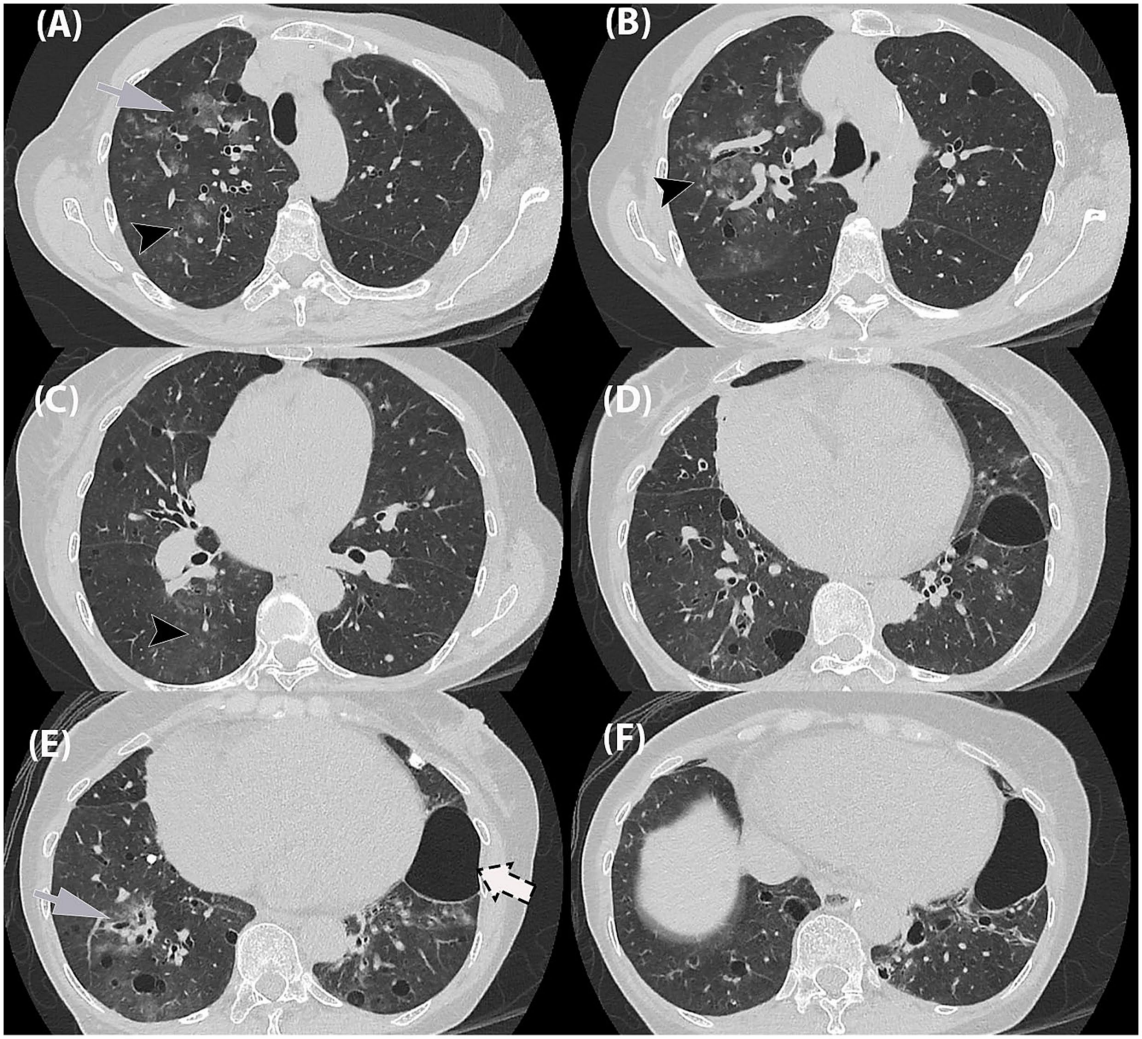
High-resolution chest tomography (HRCT) thorax in axial planes. **(A,B)** Ground-glass opacities in a bronchocentric distribution (*grey arrow*) as well as ground-glass centrilobular nodules in the right upper lobe (*black arrowhead*). **(C,D)** Similar ground-glass centrilobular nodules in right lower lobe (*black arrowhead*). **(E,F)** Ground-glass opacities in bronchocentric (*grey arrow*) distribution in bilateral lower lobes. **(A–F)** Randomly distributed thin-walled cysts of varying in bilateral lungs, **(F)** largest in left lower lobe (*dashed-arrow*).

Given the progression in radiographic findings, a high-resolution CT (HRCT) thorax was performed on Day 4. The HRCT (Figure 2) showed patchy ground-glass opacities in a peribronchovascular distribution, with diffuse peribronchial wall thickening and mucus plugging, most pronounced in the right lung. Multiple lung cysts of varying sizes were scattered throughout the lung fields, predominantly in the right lung. A large cystic lesion measuring 5.8 × 4.0 × 4.7 cm was seen in the inferior lingular segment of the left lower lobe, which could be of a pneumatocele or subpleural bullae. Additionally, a calcified pleural plaque was present at the posterior segment of the right lower lobe.

At the time of imaging, routine serology and sputum cultures were negative, thereby leaving no microbiological evidence for the patient’s acute symptoms. High-resolution CT (HRCT) was therefore central in guiding the diagnostic workup. The scan revealed airway inflammation consistent with viral infection with no radiological feature of organizing pneumonia. This posed a diagnostic challenge as the HRCT findings led to several differential diagnoses. The lung nodules were indeterminate, while the cystic lesions raised the possibility of *Pneumocystis jirovecii* pneumonia (PCP), lymphoid interstitial pneumonia (LIP), or an underlying cystic lung disease. Although acute presentation and airway inflammation favored an infective etiology, alternative diagnoses could not be excluded. A post-treatment follow-up was recommended to monitor the cystic changes.

Subsequently, a QIAstat respiratory panel was conducted via nasopharyngeal swab sample, confirming the presence of hMPV (A and B) through RT-PCR, with Ct value for hMPV A/B of 33.9. Other viral and bacterial co-infections were not detected (e.g., adenovirus, influenza, *Mycoplasma pneumoniae* etc.). Patient received supportive care and completed her course of antibiotics for atypical organisms, while antibiotics for typical bacteria were discontinued based on the viral diagnosis. Her clinical condition improved, and she was discharged with appropriate outpatient follow-up. However, no post treatment imaging follow up was recorded.

## Discussion

Human metapneumovirus (hMPV) is an enveloped, single-stranded, negative-sense RNA virus, belonging to the family Pneumoviridae within the order Mononegavirales and is phylogenetically closely related to respiratory syncytial virus (RSV) and parainfluenza viruses. Molecular characterization has delineated two major genetic lineages, designated A and B, each comprising two distinct sublineages based on antigenic and genomic analyses (2, 5–8).

hMPV is a ubiquitous respiratory pathogen infecting all age groups, with primary infection typically occurring in early childhood; seroprevalence data indicate that most children are infected by 5 years of age. Due to the induction of incomplete and transient immunity, reinfections are common throughout life. Seasonal epidemiology reveals a marked winter and spring predominance, with clinical presentations overlapping significantly with other paramyxoviruses such as RSV and influenza viruses.

The clinical spectrum ranges from mild upper respiratory tract infections (URTIs) to severe lower respiratory tract infections (LRTIs), including bronchiolitis, pneumonia, and exacerbations of chronic pulmonary diseases such as asthma and COPD. Severe disease manifestations predominantly affect pediatric populations, elderly adults, immunocompromised individuals, and those with underlying comorbidities (2, 5–7). It is primarily transmitted via respiratory secretion. The ciliated epithelial cells in the respiratory system are primarily affected, impairing mucociliary clearance (9).

Conventional viral culture is suboptimal for isolating hMPV due to its fastidious growth characteristics. Currently, nucleic-acid-based techniques (NAAT), including polymerase chain reactions (PCRs), are one of the major diagnostic approaches (9). Radiologically, hMPV primarily leads to an airway-centric infection pattern, which includes bronchial wall thickening, centrilobular nodules, peribronchial consolidation, ground-glass opacities (GGO), and nodular consolidation, often with an asymmetric bilateral multilobar involvement (1–4). In our case, typical airway-centric findings were observed. Additionally, atypical findings of multiple pulmonary cystic lesions in random distributions were also seen. These cystic lesions were of various shapes and sizes and thin walled. There was no pneumothorax.

To date, the association between hMPV and cystic lung lesions has not been reported. Pulmonary cysts, however, have been described in COVID-19 and SARS-CoV-2 infections (10–12). Generally, pulmonary cyst formation occurs through various mechanisms, including check-valve airway obstruction leading to distal airspace dilation, ischemia and necrosis of airway walls, and destruction of lung parenchyma (13). Additionally, mechanical ventilation causing cystic changes in COVID-19 patients has also been documented (12).

Pertaining to our case, these cystic changes observed may be linked to alveolar damage, as hMPV increases perivascular and peribronchiolar infiltration, triggering inflammatory responses and causing alveolar damage. This is supported by histopathological findings of acute and organizing diffuse alveolar damage (DAD), with the virus localized in bronchial epithelial cells and pneumocytes through immunohistochemistry and *in situ* hybridization (14). Other reported histopathological findings of intra-alveolar foamy macrophages, hemosiderin, and smudge cells, have also been reported, suggesting lung injury (15–17). Additionally, diffuse alveolar hemorrhage secondary to hMPV infection in adults has been documented (14, 18). However, since bronchoalveolar lavage and lung biopsy specimens are not available in this case report, the direct association between the radiologically observed pulmonary cysts and histopathological findings of DAD remains unclear. Another limitation is the availability of only a single set of CT thorax images, with no prior or follow-up images to assess the progression of the cystic lesions.

At present, there are no antivirals specifically approved for the treatment of hMPV infections. The mainstay treatment is supportive care which includes oxygen therapy and intravenous fluids. Although bronchodilators and corticosteroids are often administered as a standard practice, there is no clinical evidence confirming their effectiveness against hMPV. Ribavirin has also been explored but its clinical impact alone has yet to be ascertained. The combination of IV ribavirin and IV immunoglobulin (IVIG) has otherwise shown promising results especially in critically ill patients (9).

In conclusion, this case report underscores the possibility of hMPV infection potentially leading to the independent formation of pulmonary cysts, in addition to the typical airway-centric radiological patterns. However, the direct mechanism of multiple lung cysts during hMPV infection cannot be definitively determined. Further research is needed to fully understand the imaging dynamics associated with this virus.

## Data Availability

The original contributions presented in the study are included in the article/supplementary material, further inquiries can be directed to the corresponding author.
